# Functional outcomes of COVID-19 patients with acute ischemic stroke: A prospective, observational, single-center study in North Jordan

**DOI:** 10.1097/MD.0000000000029834

**Published:** 2022-06-30

**Authors:** Majdi Al Qawasmeh, Yaman B. Ahmed, Omar A. Nsour, Aref A. Qarqash, Sami S. Al-Horani, Ethar A. Hazaimeh, Omar F. Jbarah, Ahmed Yassin, Belal Aldabbour, Ahmed Alhusban, Khalid El-Salem

**Affiliations:** a Department of Neurosciences, Faculty of Medicine, Jordan University of Science and Technology, Irbid, Jordan; b Faculty of Medicine, Jordan University of Science and Technology, Irbid, Jordan; c Neuroscience Department, Faculty of Medicine, Islamic University of Gaza, Gaza, State of Palestine; d Department of Clinical Pharmacy, Faculty of Pharmacy, Jordan University of Science and Technology, Irbid, Jordan.

**Keywords:** acute cerebrovascular disease, COVID-19, mortality, prognosis, SARS-CoV-2, stroke

## Abstract

We assessed whether stroke severity, functional outcome, and mortality in patients with ischemic stroke differed between patients with severe acute respiratory syndrome coronavirus 2 (SARS-CoV-2) infection and those without.

We conducted a prospective, single-center cohort study in Irbid, North Jordan. All patients diagnosed with ischemic stroke and SARS-CoV-2 infection were consecutively recruited from October 15, 2020, to October 16, 2021. We recorded demographic data, vascular risk factors, National Institutes of Health Stroke Scale (NIHSS) score, stroke subtype according to the Trial of ORG 10172 in Acute Stroke Treatment Criteria (TOAST), treatments at admission, and laboratory variables for all patients. The primary endpoint was the functional outcome at 3 months assessed using the modified Rankin Score. Secondary outcomes involved in-hospital mortality and mortality at 3 months.

We included 178 patients with a mean (standard deviation) age of 67.3 (12), and more than half of the cases were males (96/178; 53.9%). Thirty-six cases were coronavirus disease 2019 (COVID-19) related and had a mean (standard deviation) age of 70 (11.5). When compared with COVID-19-negative patients, COVID-19-positive patients were more likely to have a higher median NIHSS score at baseline (6 vs 11; *P* = .043), after 72 hours (6 vs 12; *P* = .006), and at discharge (4 vs 16; *P* < .001). They were also more likely to have a higher median modified Rankin Score after 3 months of follow-up (*P* < .001). NIHSS score at admission (odds ratio = 1.387, 95% confidence interval = 1.238–1.553]; *P* < .001) predicted having an unfavorable outcome after 3 months. On the other hand, having a concomitant SARS-CoV-2 infection did not significantly impact the likelihood of unfavorable outcomes (odds ratio = 1.098, 95% confidence interval = 0.270–4.473; *P* = .896).

The finding conclude that SARS-CoV-2 infection led to an increase in both stroke severity and in-hospital mortality but had no significant impact on the likelihood of developing unfavorable outcomes.

## 1. Introduction

Since the World Health Organization proclaimed coronavirus disease 2019 (COVID-19) to be a global pandemic, it has been a significant public health burden globally.^[1]^ Neurological complications frequently occur in patients with severe acute respiratory syndrome coronavirus 2 (SARS-CoV-2) infection, affecting up to 57% of patients.^[2]^ SARS-CoV-2 has been shown to induce a state of hypercoagulability that predisposes to both arterial and venous thrombotic events.^[3]^ Stroke appears to complicate 0.2% to 5% of SARS-CoV-2 infections,^[2,4–7]^ also it remains the second leading cause of death in the world and the third leading cause of death combined with disability.^[8]^ Given early reports of an association between COVID-19 and stroke, there is a critical, unmet need to define the functional outcomes of patients with COVID-19 and stroke, which may help improve their medical care in the future. Moreover, patients with vascular risk factors associated with strokes, such as hypertension, diabetes mellitus, and previous cardiac or cerebrovascular disease are at an increased risk of mortality and morbidity.^[9,10]^

In this study, we describe the clinical characteristics and functional outcomes of stroke in patients with SARS-CoV-2 infection and evaluate the hypothesis that SARS-CoV-2 infection worsens the prognosis of patients with stroke, possibly due to harmful effects of the virus.

## 2. Methods

### 2.1. Study design and settings

We conducted a prospective, observational, single-center cohort study at King Abdullah University Hospital, the main tertiary hospital in North Jordan. The recruitment of patients began on October 15, 2020, and ended on October 16, 2021.

The ethics committee approved a waiver of consent from the patients because the study did not include any therapeutic intervention and the outcomes planned are routinely registered in patients with acute ischemic stroke. All patients diagnosed with acute ischemic stroke and SARS-CoV-2 infection were included. Patients were identified by neuroimaging studies and included in the study if the imaging showed acute ischemic stroke and classified according to the nucleic acid amplification tests as COVID-19 positive or COVID-19 negative. All patients presenting with hemorrhagic stroke or transient ischemic attack were excluded. Moreover, patients underwent a standard diagnostic evaluation, including brain imaging, intracranial and extracranial vascular imaging, and cardiac evaluation. The ischemic stroke subtype was classified according to the Trial of ORG 10172 in Acute Stroke Treatment Criteria (TOAST).^[11]^

### 2.2. Study variables

The following variables were recorded for all patients: demographic data: age and sex, vascular risk factors: history of hypertension, diabetes mellitus, hyperlipidemia, coronary artery disease, congestive heart failure, atrial fibrillation, prior stroke or transient ischemic attack, smoking, clinical data: National Institutes of Health Stroke Scale (NIHSS) score at admission and at 72 hours to observe any neurological worsening defined as an increase of ≥4 points on the NIHSS score at 72 hours, treatments at admission: statins, antiplatelet, anticoagulants, and intravenous recombinant tissue plasminogen activator, and laboratory variables (closest to the time of the stroke): cardiac troponin level, C-reactive protein (CRP), erythrocyte sedimentation rate (ESR), D-dimer level, prothrombin time, and partial thromboplastin time.

### 2.3. COVID-19 screening and diagnosis

Screening for SARS-CoV-2 was performed at first provider contact and included evaluation for recent SARS-CoV-2 exposure, history of respiratory symptoms, or chest radiographic findings. Assays for SARS-CoV-2 were performed according to World Health Organization standards. All patients had nasopharyngeal swab specimens positive for infection with SARS-CoV-2 confirmed by reverse transcription-polymerase chain reaction. The primary variable of interest was COVID-19 status (positive or negative).

### 2.4. Primary and secondary outcomes

The primary endpoint was the functional outcome at 3 months, as measured by the modified Rankin Score (mRS) evaluated through a structured telephone-based interview after obtaining oral consent at discharge. During the interview, 2 neurologists interviewed the patients or caregivers. The outcome was considered favorable when the score at 3 months was 0, 1, or 2. The secondary outcome was in-hospital mortality and mortality at 3 months. The local investigator reported the most probable cause of death.

### 2.5. Statistical analysis

All data analyses were performed using the IBM Statistical Package for the Social Sciences (SPSS) software for Windows, version 26.0. Descriptive measures included means ± standard deviations for continuous data if the normality assumption was not violated, according to the Shapiro–Wilk test, and median with first and third quartiles (Q1–Q3) if the assumption was violated. Categorical data were presented by frequencies and percentages (%).

Bivariate analyses were conducted comparing COVID-19 positive and negative patients and between the favorable and unfavorable functional outcome groups at follow-up among the COVID-19-positive cohort. Continuous data were compared using the Student *t* test in normally distributed variables and the Mann–Whitney *U* test if not normally distributed. Categorical data were compared using the χ^2^ test or the Fisher exact test if 1 cell had an expected count of less than 5.

A binary logistic regression analysis was done to identify the risk factors of having an unfavorable functional outcome in the whole cohort using the enter method. Variables included in the model were chosen based on a separate bivariate analysis, including all variables yielding a *P* value of <.1. Nagelkerke *R*^2^ was used as a measure for the goodness-of-fit. The variables in the model were checked for multicolinearity using variance inflation factor. Statistical significance was considered at a 2-sided *P* value of ≤.05.

## 3. Results

### 3.1. General sociodemographic and clinical characteristics

A total of 178 patients with radiologically proven ischemic stroke were identified in our institution during the study period, of which 36 (20.2%) were COVID-19 related, constituting 1.44% of the 2500 patients hospitalized with SARS-CoV-2 infection during the study period.

**Figure 1.**
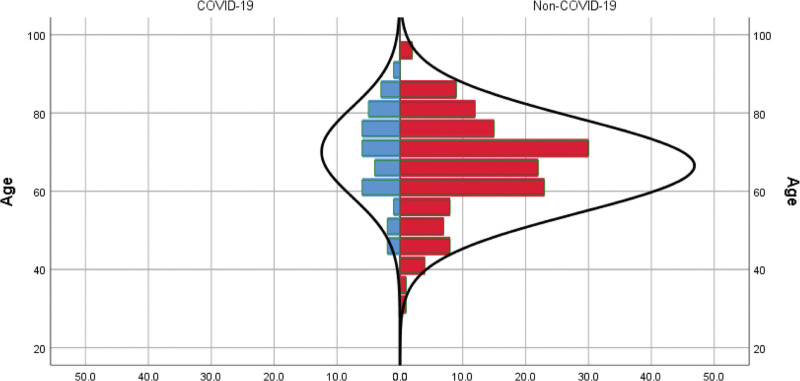
Distribution of the stroke’s patients age variable presented by a histogram. COVID-19 = coronavirus disease 2019.

**Figure 2.**
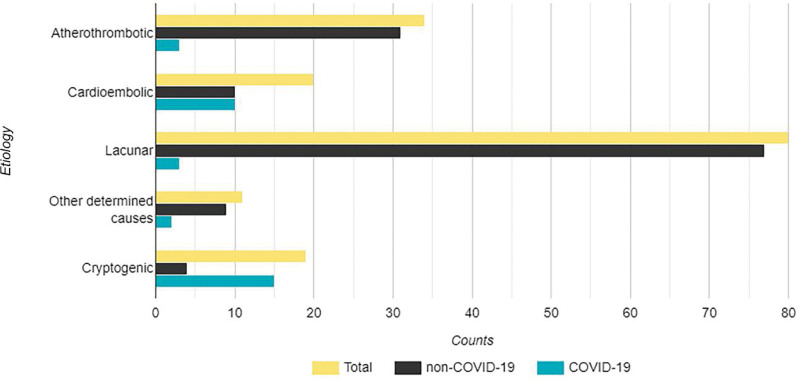
Distribution of strokes based on the etiology. COVID-19 = coronavirus disease 2019.

Overall, the patients had a mean (standard deviation) age of 67.3 (12) years on diagnosis, and more than half of the cases were males (96/178; 53.9%). Figure 1 represents a histogram of patients’ ages based on COVID-19 diagnosis. Lacunar strokes accounted for most of the cases in our cohort (80/178; 44.9%), followed by atherothrombotic (34/178; 19.1%) and cryptogenic (33/178; 18.5%) causes. Figure 2 presents the distribution of strokes based on etiology in our cohort. Patients had an overall median NIHSS score at admission and discharge of 7 (interquartile range [IQR]: 4–12) and 4 (IQR: 2–11), respectively, and an overall median mRS at discharge and 3-month follow-up of 2 (IQR: 1–3) and 3 (IQR: 1–4), respectively. Table 1 shows details of demographics, vascular risk factors, clinical data, and treatment aspects for the patients.

**Table 1:** Patients’ demographic and clinical data by COVID-19 infection status.

Variables	All subjects (n = 178)	Non-COVID-19 (n = 142)	COVID-19 (n = 36)	*P* value
Demographics				
Age (y), mean ± SD	67.3 ± 12	66.6 ± 12.1	70 ± 11.5	.121
Gender (male), n (%)	96 (54)	76 (54)	20 (56)	.674
Risk factors				
History of stroke, n (%)	33 (19)	30 (21)	3 (8)	.111
Hypertension, n (%)	141 (79)	112 (79)	29 (81)	.991
Diabetes, n (%)	108 (61)	86 (61)	22 (61)	.996
Hyperlipidemia, n (%)	63 (35)	53 (37)	10 (28)	.285
Ischemic heart disease, n (%)	50 (28)	42 (30)	8 (22)	.541
Heart failure, n (%)	24 (13)	20 (14)	4 (11)	.575
Smoking, n (%)	38 (20)	35 (25)	3 (8)	.050*
Prior treatments				
Anti-platelets, n (%)	129 (69)	103 (73)	26 (72)	.982
Anticoagulants, n (%)	23 (13)	13 (9)	10 (28)	.009*
ACE inhibitors, n (%)	67 (36)	63 (44)	4 (11)	.001*
Statins, n (%)	83 (44)	65 (46)	18 (50)	.363
NIHSS score				
Baseline NIHSS, median (IQR)	7 (4–12)	6 (4–11)	11 (5–13.5)	.043*
At 72 h, median (IQR)	7 (5–11)	6 (5–10)	12 (4.5–16)	.006*
At discharge, median (IQR)	4 (2–11)	4 (2–8)	16 (3.5–22)	<.001*
mRS				
mRS at discharge, median (IQR)	2 (1–3)	2 (1–2)	2 (1–3)	.092
mRS after 3 mo follow-up, median (IQR)	3 (1–4)	2.5 (1–4)	4 (2–6)	<.001*
Unfavorable functional outcome, n (%)	95 (53)	71 (50)	24 (67)	.073
Baseline laboratory results				
Platelets, median (IQR)	209 (165–288)	209 (164–288)	209.5 (165.5–307)	.783
C-reactive protein, median (IQR)	8.7 (3.4–50)	4.5 (3.2–11)	70.8 (27.5–146.8)	<.001*
Erythrocyte sedimentation rate, median (IQR)	21 (16–40)	19.5 (15–30)	51.5 (26–71)	<.001*
Coagulation profile at admission				
Prothrombin time (s), median (IQR)	13.8 (13–15.2)	13.5 (12.8–14.8)	15.3 (14–16.7)	<.001*
Partial thromboplastin time (s), median (IQR)	27.6 (25.6–29.4)	27.6 (25.6–29)	27.3 (24.9–29.6)	.816
D-dimer, median (IQR)	1.1 (0.5–1.5)	0.8 (0.5–1.5)	2.9 (1.6–5.5)	<.001*
Metrics				
Stroke Unit admission, n (%)	155 (87)	135 (95)	20 (56)	<.001*
ICU admission, n (%)	22 (12)	7 (5)	15 (42)	<.001*
Hemorrhagic transformation, n (%)	8 (5)	2 (1)	6 (17)	.001*
Hospital stay (d), median (IQR)	4 (3–8)	4 (3–6)	10 (4–16)	<.001*
Etiology of strokes				
Atherothrombotic, n (%)	34 (19)	31 (22)	3 (8)	<.001*
Cardioembolic, n (%)	20 (11)	10 (7)	10 (28)	
Lacunar, n (%)	80 (45)	77 (54)	3 (8)	
Other determined causes, n (%)	11 (6)	9 (6)	2 (6)	
Cryptogenic, n (%)	33 (19)	15 (11)	18 (50)	
Deaths, n (%)	19 (11)	4 (3)	15 (42)	<.001*

ACE = angiotensin-converting enzyme; COVID-19 = coronavirus disease 2019, ICU = intensive care unit, IQR = interquartile range, mRS = modified Rankin Score, NIHSS = National Institutes of Health Stroke Scale, SD = standard deviation.

* *P* value <.05.

**Figure 3.**
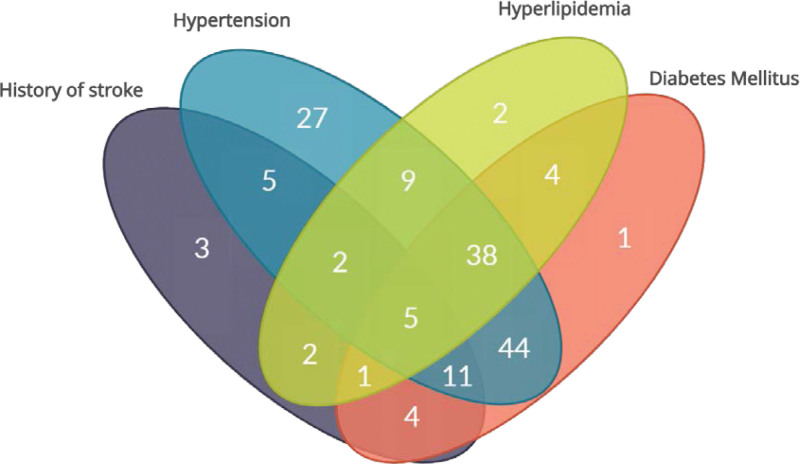
Overlapping of risk factors in the whole cohort.

Figure 3 represents a Venn diagram showing the overlap of risk factors in the whole cohort. Hypertension accounted for the most common risk factor in the cohort (141/178; 79.2%), followed by diabetes (108/178; 61%) and hyperlipidemia (63/178; 35%). A small portion of the patients also had history of stroke (33/178; 18.5%). The majority of the cohort had multiple coexisting (≥2) risk factors (125/178; 70.2%), with a minority having 1 risk factor only (33/178, 18.5%) or no risk factors at all (20/178; 11.2%).

Among patients with confirmed SARS-CoV-2 infection, 5 patients (13.9 %) presented with respiratory insufficiency (pO_2_ <60 mm Hg) during hospitalization requiring oxygen therapy. Of the 36 patients, 24 (67%) were diagnosed with COVID-19 before developing stroke symptoms, while the rest developed stroke symptoms first and were only diagnosed on hospitalization. COVID-19 symptoms experienced by our cohort included shortness of breath (50%), cough (39%), hypoxia (31%), fatigue (19%), general weakness (8%), fever (14%), and tachypnea (3%).

In our cohort, we had an overall mortality rate of 10.7% (19/178). Of which, 15 (79%) cases were in the COVID-19-positive group, and all were during hospitalization. Of those 15 patients, 10 (67%) patients died from COVID-19 complications, and 5 (33%) patients died due to stroke complications. In these 5 patients, hemorrhagic transformation with midline shift was presented during hospitalization.

### 3.2. Baseline characteristics of COVID-19-positive and COVID-19-negative patients

The detailed bivariate analysis between the COVID-19-positive and COVID-19-negative stroke patients is represented in Table 1.

The 2 groups had similar demographic and risk factor data, except that the proportion of patients with a history of smoking was significantly lower in the COVID-19 positive group (8.3% vs 24.7%; *P* = .05). Prior treatments were similar in case of antiplatelets and statins, anticoagulants were more used by the COVID-19-positive patients (27.8% vs 9.2%; *P* = .009), while ACE inhibitors were less used (11.1% vs 44.4%; *P* = .001). Laboratory results at admission showed that COVID-19-positive patients had a significantly higher median CRP levels (*P* < .001) and ESR (*P* < .001), also, coagulation profiles showed that COVID-19-positive patients had a significantly higher median prothrombin time (*P* < .001) and D-dimer levels (*P* < .001) as well. In contrast, the median partial thromboplastin time did not differ (*P* = .816). In terms of severity, COVID-19-positive patients were more likely to be admitted to the intensive care unit (ICU; 41.7% vs 4.9%; *P* < .001) and were more likely to have a higher median NIHSS score at baseline (*P* = .043), after 72 hours (*P* = .006), and at discharge (*P* < .001), and a higher median mRS at discharge (*P* = .092) and after 3 months of follow-up (*P* < .001), they were more likely to have an unfavorable functional outcome at follow-up (66.7% vs 50%; *P* = .073) as well. COVID-19-positive patients also had a higher rate of hemorrhagic transformation (16.7% vs 1.4%; *P* < .001), a higher median hospital stays (*P* < .001), and a higher mortality rate (41.7% vs 2.8%; *P* < .001).

As for the etiology of the strokes, COVID-19-positive patients had more strokes attributed to cryptogenic (50% vs 10.6%) and cardioembolic (27.8% vs 7%) causes, and less attributed to lacunar (8.3% vs 54.2%), atherothrombotic (8.3% vs 23.9%), and other determining causes (5.6% vs 6.3%; *P* < .001 for the overall comparison).

### 3.3. Follow-up of COVID-19-positive patients

The detailed bivariate analysis based on the functional outcome (favorable vs unfavorable) is presented in Table 2. Based on the 3-month follow-up, a favorable functional outcome was achieved in 33% (12/36) of the patients, and 66% had an unfavorable functional outcome. Both groups were similar for most variables, except that the median NIHSS scores were higher in the unfavorable functional outcome group at admission (*P* = .017), at 72 hours follow-up (*P* = .003), and at discharge (*P* < .001). Also, patients with favorable outcomes were less frequently admitted to the ICU (16.7% vs 54.2%; *P* = .031). Median hospital stay was also higher in patients with unfavorable outcomes, however, it was statistically insignificant (*P* = .381).

**Table 2:** COVID-19-positive patients’ demographic and clinical data stratified by the functional outcome at 3-mo follow-up.

Variables	All subjects (n = 36)	Favorable functional outcome (n = 12)	Unfavorable functional outcome (n = 24)	*P* value
Demographics				
Age (y), mean ± SD	70 ± 11.5	69.1 ± 10.2	70.5 ± 12.3	.756
Gender (male), n (%)	20 (56)	8 (67)	12 (50)	.343
Risk factors				
History of stroke, n (%)	3 (8)	2 (17)	1 (4)	.253
Hypertension, n (%)	29 (81)	9 (75)	20 (83)	.664
Diabetes, n (%)	22 (61)	8 (67)	14 (58)	.727
Hyperlipidemia, n (%)	10 (28)	3 (25)	7 (29)	1
Ischemic heart disease, n (%)	8 (22)	2 (17)	6 (25)	.691
Heart failure, n (%)	4 (11)	1 (8)	3 (13)	1
Smoking, n (%)	3 (8)	0 (0)	3 (13)	.536
Prior treatments				
Antiplatelets, n (%)	26 (72)	9 (75)	17 (71)	1
Anticoagulants, n (%)	10 (28)	4 (33)	6 (25)	.7
ACE inhibitors, n (%)	4 (11)	1 (8)	3 (13)	1
Statins, n (%)	18 (50)	7 (58)	11 (46)	.48
Stroke severity				
Baseline NIHSS score, median (IQR)	11 (5–13.5)	4.5 (3–9.5)	12 (6–14)	.017*
72 h NIHSS score, median (IQR)	12 (4.5–16)	3 (2–10)	14.5 (8.5–20)	.003*
Discharge NIHSS score, median (IQR)	16 (3.5–22)	3 (2–5)	20 (13–24)	<.001*
Admission to the stroke unit, n (%)	20 (56)	11 (92)	10 (42)	.018*
Admission to the ICU unit, n (%)	15 (42)	2 (17)	13 (54)	.031*
Hospital stay (d), median (IQR)	10 (4–16)	7.5 (3.5–15)	10 (4–18)	.381
Deaths, n (%)	15 (42)	0 (0)	15 (63)	<.001*
Laboratory findings				
Baseline O_2_ saturation, median (IQR)	92 (88.4–95)	93 (89.4–95)	92 (86.5–95)	.565
Baseline platelets count, median (IQR)	209.5 (165.5–307)	222 (176.5–295)	206 (164.5–319)	.735
Baseline CRB levels, median (IQR)	70.8 (27.5–146.8)	56 (24.9–168.8)	89.8 (34.6–146.4)	.698
Baseline ESR levels, median (IQR)	51.5 (26–71)	65 (37.5–78.5)	40 (24.5–66)	.185
Baseline D-dimer levels, median (IQR)	2.9 (1.6–5.5)	3.1 (1.5–6)	2.7 (1.6–5.2)	1
Prothrombin time, median (IQR)	15.3 (14–16.7)	15.9 (14.5–19)	15.3 (13.9–16)	.208
Partial thromboplastin time, median (IQR)	27.3 (24.9–29.6)	26.8 (25.9–29.1)	27.7 (24.1–29.9)	.849

COVID-19 = coronavirus disease 2019, CRP = C-reactive protein, ESR = erythrocyte sedimentation rate, ICU = intensive care unit, IQR = interquartile range, NIHSS = National Institutes of Health Stroke Scale, SD = standard deviation.

* *P* value <.05.

### 3.4. Predictors of unfavorable functional outcome at 3-month follow-up in the whole cohort

Presented in Table 3, a binary logistic regression analysis was performed to ascertain the effects of age, gender, SARS-CoV-2 infection, ICU admission, hemorrhagic transformation, past use of anti-platelets, NIHSS score at admission, baseline levels of platelets, CRP, and ESR, and hospital stay on the likelihood that stroke patients will have an unfavorable functional outcome versus favorable functional outcome at 3-month follow-up. The model explained 52.9% (Nagelkerke *R*^2^) of the variance in functional outcome and correctly classified 80.9% of the cases.

**Table 3:** Binary logistic regression identifying predictors of unfavorable functional outcome at 3-mo follow-up in 178 stroke patients.

Variables	No. of unfavorable outcome cases/no. of total cases (%)	Adjusted OR	95% CI	*P* value
Age (continuous)	-	1.009	0.974–1.046	.609
Gender				
Male	42/96 (44)	0.423	0.188–0.952	.038*
Female	53/82 (65)	1 (reference)	1 (reference)	Reference
COVID-19 infection				
Yes	24/36 (67)	1.098	0.270–4.473	.896
No	71/142 (50)	1 (reference)	1 (reference)	Reference
ICU unit admission				
Yes	16/22 (73)	1.348	0.260–6.994	.722
No	79/156 (51)	1 (reference)	1 (reference)	Reference
Hemorrhagic transformation				
Yes	7/8 (88)	4.426	0.358–54.727	.246
No	88/170 (52)	1 (reference)	1 (reference)	Reference
Past use of antiplatelets				
Yes	65/129 (50)	0.776	0.311–1.938	.587
No	30/49 (61)	1 (reference)	1 (reference)	Reference
Baseline findings and metrics (continuous)				
NIHSS at admission	-	1.387	1.238–1.553	<.001*
Baseline platelets levels	-	1.000	0.995–1.004	.922
Baseline CRP levels	-	0.996	0.989–1.003	.264
Baseline ESR levels	-	1.000	0.976–1.024	.999
Hospital stay	-	1.068	0.976–1.168	.152

CI = confidence interval, COVID-19 = coronavirus disease 2019, CRP = C-reactive protein, ESR = erythrocyte sedimentation rate, ICU = intensive care unit, IQR = interquartile range, NIHSS = National Institutes of Health Stroke Scale, OR = odds ratio.

* *P* value <.05.

The multivariate analysis showed that males were less likely to have an unfavorable outcome than females (OR = 0.423, 95% confidence interval [CI] = 0.188–0.952; *P* = .038), and that an increase in the NIHSS score at admission was associated with an increase in the likelihood of having unfavorable outcome (OR = 1.387, 95% CI = 1.238–1.553; *P* < .001). While having SARS-CoV-2 infection did not impact the likelihood of having an unfavorable outcome significantly (OR = 1.098, 95% CI = 0.270–4.473; *P* = .896).

## 4. Discussion

This single-center prospective study reports key demographic and clinical characteristics of patients who develop acute ischemic stroke and concomitant SARS-CoV-2 infection in King Abdullah University Hospital. The observed rate of imaging-confirmed acute ischemic stroke in hospitalized patients with COVID-19 of 1.44% was comparable with prior reports.^[2,4–7]^ However, the initial estimates in Wuhan suggested a higher proportion of ≈5% of acute ischemic stroke among patients hospitalized with SARS-CoV-2 infection.^[2]^ Other cohort studies have reported that the proportion of patients with acute ischemic stroke may range between 1% and 3% among hospitalized COVID-19 patients receiving thromboprophylaxis.^[12–14]^ Another recent retrospective study by Merkler et al^[15]^ reported that 0.9% of 3556 hospitalized patients with COVID-19 had an acute ischemic stroke.

Our findings suggest that most of the COVID-19 patients who develop acute ischemic stroke have preexisting vascular risk factors for large vessel atherosclerosis, small vessel disease, and cardioembolism. These findings may differ from the earlier observations from smaller case series that suggested that patients with SARS-CoV-2 infection who develop acute ischemic stroke were younger and without preexisting cardiovascular risk factors.^[5,16,17]^ Other studies suggested that, even if SARS-CoV-2 infection was a predisposing factor, the risk was mainly seen in those who were already at risk for acute ischemic stroke due to other vascular risk factors.^[18–20]^ In agreement with this assumption, patients in our study were typical of a stroke cohort, with a mean age of 67.3 years, a predominance of males (55.8%), and a high proportion of patients with varied vascular risk factors. Moreover, the demographic and clinical features were comparable to an earlier cohort from our center with a mean age of 66.5 years, a predominance of males (53.52%) and a high proportion of patients with vascular risk factors.^[21]^ SARS-CoV-2 infection can act as a trigger of conventional stroke causes.^[22]^ Like other viral infections, SARS-CoV-2 infection may increase the risk of stroke.^[22]^ The higher proportion of strokes of cryptogenic nature observed in our study may be explained by the different COVID-19-related stroke pathogeneses described.^[22]^ Cytokine storm, prothrombotic state, antiphospholipid syndrome, myocardial injury, arrhythmias, and endothelial activation and dysfunction have been proposed.^[22–26]^

We hypothesized that patients with acute ischemic stroke and SARS-CoV-2 infection would have a worse prognosis. Stroke severity measured by the NIHSS score was high in our cohort. Moreover, the NIHSS score remained higher at 72 hours, reflecting the persistence of a worse neurological deficit. In our cohort, we observed a low proportion of favorable functional outcomes (33%) and high in-hospital mortality (42%). A recent prospective study conducted by Martí-Fàbregas et al^[27]^ also found a lower proportion of favorable functional outcomes in patients with COVID-19 than in patients without (33.7% vs 47%). It is challenging to determine if these differences in outcome were due to worse stroke severity in COVID-19 settings or due to difficulties in stroke care and rehabilitation in patients diagnosed with SARS-CoV-2 infection. Based on our data, we believe that both scenarios should be considered. Although the difference in functional outcome observed in our study was not statistically significant, we reported well-known predictors of functional outcome. Baseline NIHSS, ICU admission, vascular risk factors were all associated with poor functional outcomes in our patients. This agrees with other prognostic studies.^[20,28–30]^ Moreover, a recent retrospective study conducted by Qin et al^[20]^ reported that a prior stroke in patients with SARS-CoV-2 infection was independently associated with severe clinical symptoms and poorer outcomes than those without a history of stroke after a propensity-matched analysis.^[20]^ It is worth mentioning that our results show that males were less likely to have an unfavorable outcome than females, contrary to previous studies reporting a worse prognosis in males.^[31,32]^ However, this finding could not be properly investigated due to our low sample size. Due to that, a study on a larger sample size should investigate sex disparities in COVID-19 severity and functional outcome in patients with ischemic stroke.

Regarding mortality, our findings agree with recently published studies on patients with stroke which reported that infection with COVID-19 was associated with higher mortality rates.^[5,7,27]^ About 67% (10/15) of deaths in our study were due to COVID-19 complications. Several aspects related to the SARS-CoV-2 infection may explain our findings, including the respiratory distress, multiorgan failure, and elevation in serum marker of inflammation and fibrin activation, all of which have been shown to increase mortality and morbidity rates in patients with acute ischemic stroke.^[2,5,17,18,20]^ Besides the cardiovascular risk, Trifan et al^[31]^ reported that the interplay of aging, comorbidities, and COVID-19 respiratory symptoms is associated with higher mortality rates compared with young patients without any risk factors or comorbidities.

The pandemic has impacted the ability of health systems to care for patients with stroke and other medical emergencies. These obstacles to routine stroke care in Jordan may explain in part the poor functional and clinical outcomes observed. This has also been reported by authors in other countries.^[33–38]^ Many institutions are attempting to balance the benefits of rapid, structured neurological evaluations for patients with SARS-CoV-2 infection experiencing new neurological symptoms with the risk of exposing medical staff to infection.^[36]^ There are multiple implications for clinical and stroke care in Jordan. First, we recommend testing all patients with suspected stroke for SARS-CoV-2 infection at admission, provided local laboratory capacity is sufficient.^[39]^ This recommendation is based on the observed evidence that many patients with stroke may test positive even when systemic signs and symptoms of infection are absent.^[5,15,40,41]^ This ensures that appropriate isolation precautions are implemented for patients testing positive and allows early recognition of COVID-19 symptoms. Additionally, acute SARS-CoV-2 infection in a patient with stroke has further implications for the underlying mechanism of stroke, choice of optimal therapy, and the long-term risk of recurrence. Second, management of ischemic stroke patients with SARS-CoV-2 infection should follow the same standards of care as for patients without COVID-19,^[42]^ but with necessary precautions related to infection control.^[43–45]^ Furthermore, early initiation of acute antithrombotic therapy is probably reasonable, given the high thrombotic risk seen in patients with COVID-19.^[46]^ However, it should be noted that 5 patients in our cohort developed hemorrhagic transformation during hospitalization. This complication could be attributed to the interplay of endothelial dysfunction compounded with anticoagulant treatment and preexisting comorbidities. In a report of 3824 hospitalized patients with COVID-19, intracerebral hemorrhage was reported in 33 patients (0.9%).^[47]^ Based on the radiologic pattern, the authors inferred that approximately three-quarters of these may have resulted from the hemorrhagic transformation of ischemic stroke. Another study on 278 hospitalized patients with COVID-19 had reported intracerebral hemorrhage in 10 patients. In both studies, most of the patients with intracerebral hemorrhage had been treated with a full dose of anticoagulants.^[47,48]^ Due to that, an assessment of the severity of systemic illness, presence of other thrombotic events, and bleeding risk must be considered before initiation of optimal antithrombotic therapy.

Our findings should be interpreted with caution in the context of several limitations. First, our study was a relatively small, single-center, observational study with potential for selection bias. Second, we did not have complete comprehensive workups for some patients who died during hospitalization, and therefore they could have been prematurely classified as “cryptogenic” and may be related to another undiagnosed mechanism. This likely contributed to an increased prevalence of cryptogenic strokes in patients with SARS-CoV-2 infection in our study. Finally, our study may not be fully representative of all stroke patients in our hospital; patients who are critically ill may not be diagnosed with stroke due to impaired level of consciousness or confounding systemic illnesses. Despite these limitations, our study is one of the first international reports on the characteristics and functional outcome of ischemic stroke with concomitant SARS-CoV-2 infection. We believe that this study will help improve the care of patients with stroke and concomitant SARS-CoV-2 infection.

## 4.5. Conclusion

This study illustrates the intricate relationship between COVID-19 and acute ischemic strokes. Patients with ischemic strokes occurring with concomitant SARS-CoV-2 infection have more severe strokes and unfavorable functional outcomes after 3 months of follow-up. It is important to emphasize that, despite the higher mortality in our cohort, SARS-CoV-2 infection did not impact the likelihood of developing unfavorable outcomes. COVID-19 patients who developed acute ischemic stroke had preexisting cardiovascular risk factors. Thus, these patients were at a high risk of developing a stroke. The pathophysiology behind strokes occurring in COVID-19 patients remains not fully understood. Therefore, future controlled, prospective international studies with a higher sample size are needed to establish an evidence-based approach to stroke occurring in COVID-19 patients and elaborate more on their outcomes.

### Author contributions

Conceptualization: MA, YBA, KE, AA, AY.

Former analysis: AQ, SA, YBA.

Investigation: MA, YBA, ON, EH, KE, AY.

Methodology: MA, YBA, ON, EH, KE, AA, AY.

Resources: MA, YBA, OA, EH, OJ.

Supervision: MA, YBA, KE, AA, EA, AY.

Validation: MA, YBA, KE.

Writing – original draft: MA, YBA, KE.

Writing – editing: MA, YBA, ON, SA, AQ, EH, OJ, BA.
